# Breastfeeding self-efficacy status and determinant factors among postnatal women in public hospitals of the Gurage Zone, Central Ethiopia: a mixed study design

**DOI:** 10.1017/S1463423626101224

**Published:** 2026-04-27

**Authors:** Mangistu Abera, Megertu Obsa Gelmesa, Ayele Sahile Abdo, Keyredin Nuriye Metebo, Aynalem Belay, Aberash Beyene Derribow

**Affiliations:** 1 Midwifery, Wolkite University, Ethiopia; 2 Wolkite University, Ethiopia

**Keywords:** breastfeeding, Ethiopia, postnatal women, self-efficacy

## Abstract

**Background:**

Breastfeeding self-efficacy is a mother’s confidence in her ability to breastfeed, influencing her decision to do so. Across the world, due to low breastfeeding self-efficacy and limited attention to breastfeeding practices expose mothers and infants to adverse health outcomes and poor mother–child bonding and development. Most previous studies focused on breastfeeding knowledge, but there is limited research on breastfeeding self-efficacy and its associated factors. Thus, this study aimed to assess breastfeeding self-efficacy status and determinant factors among postnatal women in Gurage Zone public hospitals, Central Ethiopia.

**Methods:**

A facility-based mixed-method cross-sectional study was conducted among 422 systematically selected women in Gurage Zone public hospitals, Central Ethiopia, from February 15 to March 15, 2025. Data were collected using a pre-tested interviewer-administered questionnaire, entered into Epi-Data 4.1, and analyzed using SPSS 26. Four focus group discussions were conducted with purposive sampling and analyzed thematically. Binary logistic regression identified factors associated with breastfeeding self-efficacy at P < 0.05.

**Results:**

In this study, the overall breastfeeding self-efficacy status was 51.3% (95% CI: 47, 56). Women’s primary educational attainment (AOR = 1.97; 95%CI:1.01,3.83), secondary and above educational attainment (AOR = 3.30; 95%CI: 1.87,5.85), ANC contacts (AOR = 2.24; 95%CI:1.37,3.63), breastfeeding experience (AOR = 3.59; 95%CI:2.10,6.13), moderate perceived social support (AOR = 2.96; 95%CI:1.47,5.55), and high perceived social support (AOR = 3.23; 95%CI:2.02,6.59) were significantly associated with breastfeeding self-efficacy.

**Conclusion:**

In this study, 48.7% lacked breastfeeding confidence. Educational status, ANC contact, breastfeeding experience, and perceived social support were associated with breastfeeding self-efficacy. Therefore, strengthening health education and counseling is needed to improve postpartum women’s breastfeeding confidence..

## Introduction

Breastfeeding self-efficacy is a mother’s confidence in her ability to breastfeed her infant rather than her actual practice that improves breastfeeding outcomes (Dennis, 1999; McGovern *et al*., 2024).

Globally, over two-thirds of child deaths in the first year of life are often related to poor exclusive breastfeeding practices (UNICEF, 2017). Around 41% of global deaths due to poor breastfeeding practices were from Sub-Saharan Africa (Woldie, 2014; Saqib and Qazi, 2018). In Ethiopia, 270,000 malnutrition-related deaths among children under the age of five were due to a lack of exclusive breastfeeding (Jarso *et al*., 2015). The evidence revealed that approximately 21.9% of women were introduced to additional foods before six months (Kasahun *et al*., 2017). To tackle the low breastfeeding rates, breastfeeding self-efficacy (BFSE) should be an important determinant of breastfeeding initiation, duration, and patterns (Kingston *et al*., 2007). According to the WHO, breastfeeding self-efficacy is modifiable, has positive results on exclusive breastfeeding (WHO & UNICEF, 2017), and reduces the number of women who stop breastfeeding, especially in the first few months (Borona *et al*., 2023; McGovern *et al*., 2024).

Across the world, the magnitude of breastfeeding self-efficacy ranged from 38.8% to 90.2% (Mirghafourvand *et al*., 2018; Hemiyanty *et al*., 2022; Vaithilingan and Johnson, 2023). The evidence indicated that high levels of breastfeeding self-efficacy increased breastfeeding initiation and duration, improved problem-solving, and reduced stress and anxiety around breastfeeding, promoting maternal well-being (Awaliyah *et al*., 2019; Brani *et al*., 2024).

Low self-efficacy can precipitate the early cessation of breastfeeding, with mothers perceiving breastfeeding as painful and challenging, leading to negative experiences and feelings of guilt (De Sá Vieira Abuchaim *et al*., 2016; Titaley *et al*., 2021). According to the studies, more than half of women with children under 6 months have low breastfeeding self-efficacy (Economou *et al*., 2021; Titaley *et al*., 2021). A mother who had low breastfeeding confidence in their ability was 2 to 3 times more likely to stop breastfeeding earlier than the recommended period (Awaliyah *et al*., 2019).

The various studies showed that education level, working outside the home, receiving breastfeeding advice, young maternal age, income, number of pregnancies, lack of breastfeeding experience, breastfeeding problems, cesarean section delivery, and social and partner support were associated with breastfeeding self-efficacy (Schwartz *et al*., 2002; Taveras *et al*., 2003; O’Brien *et al*.,, 2008; Pelt *et al*., 2020; Topuz *et al*., 2021).

The evidence revealed that the lack of breastfeeding self-efficacy has several consequences, such as early cessation of breastfeeding, reduced lactation, motivation & low mother-to-baby bonding, reduced mother’s mood, and increased level of her anxiety and interference with the cognitive abilities, negative effects on maternal feeling and performance (Glassman *et al*., 2014; De Sá Vieira Abuchaim *et al*., 2016; Maleki-Saghooni *et al*., 2017).

Moreover, breastfeeding self-efficacy is crucial to preventing early weaning, which leads to infant morbidity and mortality, particularly in developing countries that do not have adequate medical equipment and resources (Hinic, 2016; Awaliyah *et al*., 2019). Furthermore, the evidence revealed that promoting breastfeeding requires addressing prevalent myths, knowledge gaps, and awareness of certain aspects of breastfeeding (Kumari and Jain, 2024). It is a critical factor influencing whether mothers initiate breastfeeding, persist through challenges, and achieve their breastfeeding goals (Zeng *et al*., 2024). Again, as far as searching capacity is concerned, the principal investigator could not find evidence that the study was conducted in a study setting on breastfeeding self-efficacy and its determinant factors among postnatal mothers. Therefore, this study aimed to assess breastfeeding self-efficacy status and determinant factors among postnatal women in Gurage Zone public hospitals, Central Ethiopia.

## Methods and materials

### Study area and period

In central Ethiopia, Gurage is an administrative Zone. Wolkite Town is the capital of the Gurage Zone. The distance between the Gurage zone and Addis Ababa, the capital of Ethiopia, is 153 km southwest. The Gurage zone has 1,279,646 residents, overall, 657,568 of whom are women and 622078 of whom are men, according to the 2007 national household census. (Ethiopia, 2007) It has 14 districts and 5 town administrations. The Zone has four hospitals and 72 health centers. All hospitals provided maternal health care services to mothers, including antenatal care, labor and delivery, and postpartum care. The study was conducted from February 15 to March 15, 2025.

### Study design

A multicenter facility-based cross-sectional study with a concurrent mixed approach was employed from February 15 to March 15, 2025.

### Source population

All postnatal women in Gurage Zone public hospitals, Central Ethiopia.

### Study population

All postnatal women in public hospitals during the data collection period.

### Eligibility criteria

#### Inclusion criteria

All postnatal women who received postnatal care and were willing to participate during the data collection period.

#### Exclusion criteria

Postnatal women who have severe medical illness, breast complications, complementary feeding, and have known mental illness were excluded.

### Sample size determinations

The sample size was determined using a single population proportion formula by considering a population proportion of 50% with a 95% confidence interval and a 0.05 margin of error.

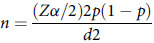




With the assumptions of 95% CI, estimated breastfeeding self-efficacy status among postnatal women at 50% (*P* = 0.5), the formula yields 384. To calculate the final sample size, use the single population proportion formula: *n* = 1.96.2 (0.5) (1–0.5)/(0.05)2 = 384.16 ≈ 384. Adding a 10% non-response rate leads to a final sample size of 422.

For the qualitative data, a thematic qualitative approach was implemented. A total of four focus group discussions were conducted. Each focus group consisted of six mothers. The qualitative sample size was determined using the principle of data saturation.

### Sampling techniques and procedures

For quantitative data, four public hospitals were founded in the Gurage Zone. All four hospitals were included. The average number of clients who were followed up on postnatal care at each facility over two months was approximately 860 (*N*). The number of respondents in each hospital was proportionally allocated based on the average number of mothers who received maternity care at each hospital. Following this, systematic random sampling was used to select the study participants in each hospital, as K = N/*n* = 860/422 = two (2). The interview was conducted after selecting the first respondent randomly by lottery methods from the women who attended the maternity care unit on the first day of data collection, and each respondent corresponding to the skip interval was chosen after that.

For the qualitative component, postnatal mothers were purposively selected from the postnatal unit through maternity healthcare providers to participate. Data were collected through four focus group discussions (FGDs), each consisting of six mothers, to explore in depth individual experiences regarding breastfeeding self-efficacy. Data collection continued until saturation was achieved, and a few additional discussions were conducted to confirm that no new information was emerging from respondents across the focus group discussions. All FGDs were conducted in facility-based venues that were convenient, neutral, and ensured privacy and confidentiality for the participants.

### Operational definitions

#### Exclusive breastfeeding

If a mother feeds her infant exclusively breast milk for the entire 6 months, she should not feed her infant any other liquids or solids other than vitamins, mineral supplements, or medications.

#### Breastfeeding self-efficacy

Breastfeeding self-efficacy refers to a mother’s confidence in her capacity to breastfeed her baby (Dennis, 1999; McGovern *et al*., 2024).

#### Perceived social support

refers to an individual’s belief that emotional, informational, and practical help will be available from their social network, such as family, friends, or other significant persons, when needed (Scarapicchia *et al*., 2017).

### Study variable

#### Dependent variable

Breastfeeding self-efficacy status

### Independent variables

#### Sociodemographic characteristics

Maternal age, residence, religion, maternal education status, marital status, occupation, husband’s educational status, husband’s occupation, and income

#### Obstetrics-relation questions

Parity, ANC follow-up, pregnancy intention, gestational age, number of children, mode of delivery, sex of the current baby

#### Personal related factors

Perceived social support (friends support, family support, other significant supports), having breastfeeding information, breastfeeding experience, and plan to breastfeed

### Data collection tools and measurements

The data collection tool was adapted from similar research on the outcome of interest and used for collecting data (Uyar Hazar and Uzar Akça, 2018; Gizaw *et al*., 2022; Abageda *et al*., 2024; Brani *et al*., 2024). The questionnaire was prepared in an English version. Then, it was translated into the Amharic version and then to the Guragigna language by a language expert. After, it was translated back to the English version to check its consistency. Postpartum mothers’ sociodemographic information, obstetrics-related factors, personal-related factors, and Breastfeeding Self-Efficacy Scale Short Form (BSES-SF) were included in the tool. To ensure reliability and consistency, the tool was checked by a panel of experts, and a reliability test was conducted. The data was collected by trained nine diploma nurses, and four BSc nurses supervised by holding nurses who are fluent in the local language.

### Regarding BFSE measurement

Breastfeeding self-efficacy was measured using the Breastfeeding Self-Efficacy Scale Short Form, which was published in different reputable journals (Uyar Hazar and Uzar Akça, 2018; Gizaw *et al*., 2022; Abageda *et al*., 2024; Brani *et al*., 2024). We used the BSES-SF, a 14-item questionnaire with a five-point Likert scale developed to measure breastfeeding confidence in Amharic that was translated from validated English questionnaires from various studies, to assess the mother’s self-efficacy in her ability to breastfeed. All of the items are preceded by the phrase ‘I can always’ and are anchored with a 5-point Likert scale, with one indicating no confidence at all and five indicating very confident.

The scale’s score was calculated by taking the average of all items. A higher total score indicates that the mother is more confident in her ability to breastfeed. All items are presented positively, and scores are summed to produce a range from 14 to 70. In this study, BFSE-SF was operationalized as participants with scores equal to or more than the mean were considered to have high breastfeeding self-efficacy, and those with scores below the mean were considered to have low breastfeeding self-efficacy. A higher total score indicates that the mother is more confident in her ability to breastfeed (Poorshaban *et al*., 2017; Abageda *et al*., 2024). In this study, a Cronbach’s α value of BFSE-S is 0.93.

#### Regarding social support measurement

In this study, perceived social support was assessed using the tool developed by Zimet *et al*., 1988, the multidimensional perceived social support scale (MSPSS), which consists of 12 items with three subscales: family, friend, and significant others (Zimet *et al*., 1988). The lowest score that can be obtained from the subscales is 4, and the highest score is 28. For the whole scale, the lowest possible score is 12, and the highest is 84. Based on these scores, perceived social support can be categorized into low, moderate, and high levels. A high score indicates high perceived social support. The original reliability test showed a Cronbach’s α value of 0.88, and in this study, it was 0.89 (Konukbay *et al*., 2024).

Qualitative data were collected using FGDs to supplement the quantitative data. The focus group discussion guides were structured into sections covering key thematic areas related to breastfeeding self-efficacy. Each section included open-ended questions with probes to explore participants’ experiences in detail. The FGDs lasted 90 minutes each to explore in detail breastfeeding self-efficacy. Four BSc nurses with prior experience in qualitative data collection conducted the focus group discussions. Each session was audio-recorded after obtaining written informed consent from the participants. The place and time of discussion were selected to be convenient for the study participants. The principal investigator and supervisors selected the participants, and the discussions were conducted until saturation of ideas occurred. The supervisors moderated the discussions, while the data collectors took notes and recorded all the information from the focus group discussions.

### Data quality control

To ensure data quality, language experts translated the questionnaire into Amharic and Guragigna. The tool was pre-tested on 21 postpartum mothers at Gubre Health Center before actual data collection to ensure clarity, consistency, cultural relevance, and completeness. Data collectors and the supervisor received two days of training on the study’s objectives, questionnaire clarification, sampling strategy, and data collection process and supervision. The principal investigator and supervisor guided, facilitated, and checked data completeness, ensuring the overall activities were effectively managed throughout the data collection process. Data was coded, entered, and rechecked during data entry into the computer software before analysis. Simple frequencies and a box plot were used to look for missing values and outliers, respectively.

For qualitative data, field notes were taken during the focus group discussions, and a tape recorder was used to support accurate transcription. After each session, the recordings and notes were reviewed to ensure completeness, consistency, and clarity of data. Furthermore, the trustworthiness of the study was ensured following Guba and Lincoln’s criteria (Guba and Lincoln 1985). Credibility was enhanced through member checking and triangulation, dependability through an audit trail, confirmability through reflexive notes, and transferability by providing detailed descriptions of the study objectives.

### Statistical analysis

After ensuring the questionnaire’s completeness, the data were checked, coded, and entered into the EPI data 4.1 statistical packages before being analyzed with SPSS version 26 software. Binary and multivariate logistic regressions were used to identify the relationships between predictors and outcome, and variables with a p-value of less than 0.25 at a 95% confidence interval were considered for multivariable logistic regression analysis. This approach is widely recommended in the literature to avoid excluding potentially important variables that may not show a strong association in bivariable analysis but could be significant in the multivariable model after adjusting for confounders. An odds ratio with a 95% confidence interval was used to determine the association between dependent and independent variables. Significant associations are defined as *p*-values <0.05.

Multicollinearity was checked to see the linear correlation among the associated independent variables by using the variance inflation factor (VIF) and standard error. A VIF of >10 or a standard error of >2 was considered suggestive of multicollinearity. For all independent variables, the multicollinearity effect was checked by co-linearity diagnostic statistics via VIF and tolerance test with a maximum value of 1.20 and a minimum value of 80.8%, respectively. In multivariable analysis, the multivariable logistic regression model was used to control the confounders. The Hosmer–Lemeshow goodness-of-fit test was done to check for model fitness with a *p*-value of 0.775, which indicates the model was fitted. Adjusted Odds Ratio (AOR) with a 95% confidence interval was estimated to demonstrate the strength of the association between the independent variables, after controlling for the effects of confounders. The results were considered statistically significant at a *P*-value < 0.05.

For qualitative data, thematic analysis approaches were conducted. The audio-taped qualitative data were transcribed verbatim and translated into English. The transcripts were read repeatedly by the principal investigator and research team to gain familiarity with the data and to identify initial patterns. Then, codes or terms were identified and tallied to develop categories, which were later used to establish themes based on the objectives of the study. Proper coding and categorization of data were maintained to ensure the quality of the analysis. To ensure reliability and validity, coding and theme development were independently performed and then compared; discrepancies were discussed and resolved through consensus. Furthermore, findings were presented to sampled participants to confirm that the themes accurately reflected their breastfeeding self-efficacy and its determinants. Audit trails were maintained throughout the analysis to enhance the transparency of the research process. Finally, the findings were triangulated with the quantitative results by comparing survey outcomes with themes emerging from focus group discussions, allowing the study to elaborate, complement, and contextualize the results on breastfeeding self-efficacy.

## Results

Among a total sample of 422 study participants, 419 were interviewed and gave a response rate of 99.3%, and the results were presented as follows under subheadings.

### Sociodemographic characteristics of the participants

More than half, 259 (61.8%), of the participants were in the age group 25–34 years. The mean age of the respondents was 29.83 (± 6.25 SD) years. Two hundred seventy-seven (66.1%) of the study participants were urban residents. Nearly half of the study participants, 177 (42.2%), were followers of the Muslim religion.

Among the study participants, 412 (98.3%) were married. More than half of the women, 288 (68.7%), attended formal education, and 152 (36.3%) of them reported that their husbands’ educational status was secondary and above. One hundred seventy-four (41.5%) of the women were housewives, and 45.5% of them reported that their husbands’ occupation was merchant (Table 1).

**Table 1.** Socio-demographic characteristics of the study participants in the Gurage Zone public hospitals, Central Ethiopia, 2025 (*n* = 419)

Variables		Frequency (*n* = 419)	Percentage (%)
Age of the women [in years]	15–24	61	14.6
	25–34	259	61.8
	35–49	99	23.6
The mean ±SD age of the respondents was 29.83 ± 6.25			
Residence	Rural	142	33.9
	Urban	277	66.1
Marital status	Un married	7	1.7
	Married	412	98.3
Religion	Orthodox	152	36.3
	Muslim	177	42.2
	Protestant	57	13.6
	Catholic	33	7.9
Women’s educational status	No formal education	131	31.3
	Primary education	94	22.4
	Secondary and above	194	46.3
Women’s occupational status	House wife	174	41.5
	Self-employee	123	29.4
	Government employee	122	29.1
Husband’s educational status	No formal education	113	27.4
	Primary education	147	35.6
	Secondary and above	152	37
Husband’s occupational status	Farmer	63	15.0
	Merchant	190	45.5
	Government employee	120	28.6
	Daily labors	39	9.3
Income	500–2500 ETB	27	6.4
	2501–5000 ETB	92	22.0
	> 5000 ETB	300	71.6

*ETB: Ethiopian birr*.

### Obstetrics characteristics of the participants

Of the total study participants, 371 (88.5%) attended antenatal care; from these, 178 (48%) women had eight or more antenatal care contacts. In addition, two hundred thirty-three (55.6%) of the study participants were multiparous. Regarding pregnancy intention, 300 (71.6%) had an intended pregnancy. Furthermore, 233 (55.6%) had previous breastfeeding experiences. Moreover, among the total study participants, 257 (61.3%) had a plan to breastfeed their newborn up to 2 years (Table 2).

**Table 2.** Obstetrics characteristics of the study participants in the Gurage Zone public hospitals, Central Ethiopia, 2025 (*n* = 419)

Variables		Frequency (*n* = 419)	Percentage (%)
Parity	Primiparous	186	44.4
	Multiparous	233	55.6
Attend ANC	Yes	371	88.5
	No	48	11.5
Number of ANC contacts	<8 contact	193	52.0
	≥ 8 contacts	178	48.0
Breastfeeding information during ANC	Yes	247	58.9
	No	124	29.6
Current pregnancy intention	Unintended	119	28.4
	Intended	300	71.6
GA of the recent baby	Preterm	23	5.5
	Term	370	88.3
	Postterm	26	6.2
Mode of delivery	Cesarean section	93	22.2
	Instrumental	46	11.0
	SVD	280	66.8
Sex of the recent baby	Male	177	42.2
	Female	242	57.8
Number of children	1	186	44.4
	2–4	174	41.5
	≥5	59	14.1
BF information after delivery	No	166	39.6
	Yes	253	60.4
BF experience	No	186	44.4
	Yes	233	55.6
Plan to breastfeed	Up to 6 months	38	9.1
	Up to 1 year	124	29.6
	Up to 2 years	257	61.3

*Note:* ANC: Antenatal care, BF: Breastfeeding, SVD: Spontaneous Vaginal Delivery, GA: Gestational age.

**Figure 1.**
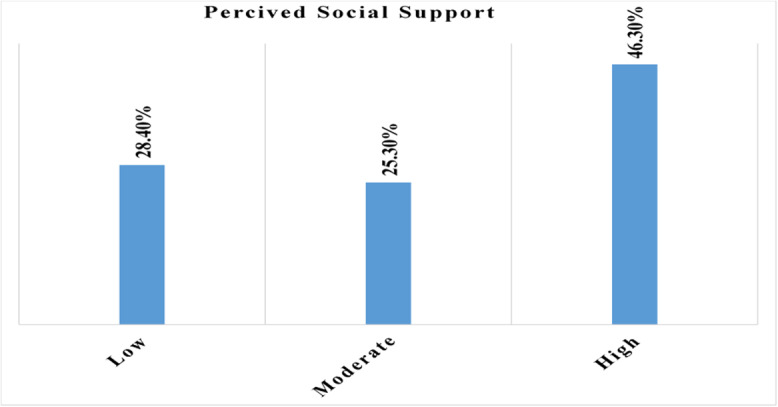
The proportion of perceived social support among postnatal women in Gurage Zone public hospitals, Central Ethiopia, 2025 (*n* = 419).

Of 419 study participants, 194 (46.3%) had high perceived social support regarding breastfeeding self-efficacy (Figure 1), and 215 (51.3%) of the study participants had support from their husbands during breastfeeding their babies.

### Breastfeeding self-efficacy status of the postnatal women

**Figure 2.**
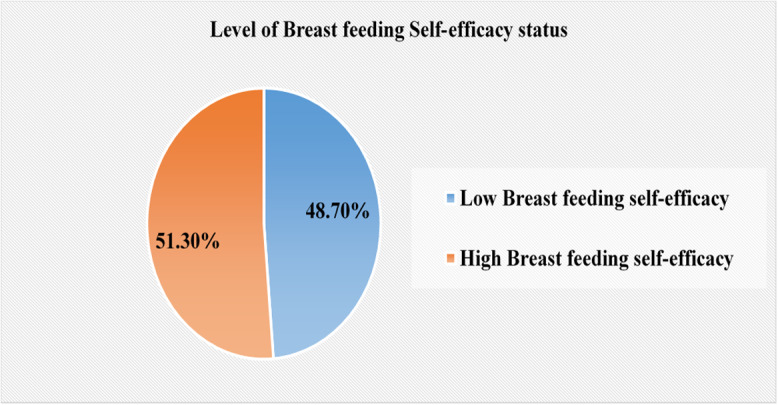
The overall breastfeeding self-efficacy status among postnatal women in Gurage Zone public hospitals, Central Ethiopia, 2025 (*n* = 419).

In this study, the overall breastfeeding self-efficacy status was 51.3 % (95% CI: 47, 56) (Figure 2).

### Qualitative findings regarding breastfeeding self-efficacy status

The qualitative findings from the focus group discussions data revealed three interrelated themes and several subthemes describing how postnatal women and their communities experience breastfeeding self-efficacy. These themes are low breastfeeding self-efficacy among postnatal mothers, determinants undermining breastfeeding confidence, and determinants enhancing breastfeeding confidence.

### Low breastfeeding self-efficacy among postnatal mothers

The majority of the discussants reported that their confidence in breastfeeding their babies after birth was not as expected. This shows a lack of understanding of the benefit of confidently breastfeeding their babies, even during challenges.

Primiparous mothers expressed further breastfeeding challenges. First-time mothers often reported low confidence in their ability to breastfeed, particularly when family advice conflicted with health recommendations.

*“This is my first baby, and I have no experience breastfeeding confidently. I wanted support, but my husband was too busy… my mother-in-law told me to feed the baby butter in addition to breast milk. I was not confident in this challenge, so I decided to give butter as my mother-in-law recommended, even though I know complementary feeding is not advised up to six months” (A 21-year-old primiparous mother, FGD).*

These reflect mothers’, particularly primiparous mothers’, challenges of breastfeeding confidence.

### Determinants undermining breastfeeding confidence

Postnatal mothers described several factors that reduced their confidence in breastfeeding. Women’s work responsibilities and limited social support significantly undermined some mothers’ breastfeeding confidence.

*“This is my second baby. I do not feel confident about breastfeeding because I am busy with work during the day, and at night, I have no support; rather, I must cook for my first child, my husband, and my family. Unlike my best friend, I do not plan to breastfeed my baby for up to two years. Even though I know it may be wrong, I have a plan to stop breastfeeding before six months.” (24-year-old FGD participant).*

This indicated how work responsibilities and social support significantly influence the mothers’ breastfeeding self-confidence.

Community beliefs and myths also influence breastfeeding self-efficacy. In this study, the majority of the mothers described community myths around breastfeeding created feelings of shame and fear, leading to early cessation of breastfeeding their babies.

*A 31-year-old FGD participant said, “This is my third baby. I have a plan to start additional food after three months because I am not confident breastfeeding in front of other people, even my extended family. In our community, there is a belief that bad spirits can affect breast milk production and babies’ comfort. Because of this, I do not feel confident breastfeeding when people are around me.”*

These beliefs and myths make the mothers emotionally not ready to breastfeed their babies.

During the focus group discussion, some mothers raised the gender preference and mistreatment as barriers to breastfeeding confidence.

*“This is my six baby. My husband and my mother-in-law were expecting a boy, but my baby was a girl. Because of her sex, they hate me and interrupt me while I am breastfeeding. Even the community does not like me, because they believe that at least one baby should be male. Because of this, I fear that my baby’s development is less than expected for her age, and I am not confident in breastfeeding my baby girl.” (A 39-year-old FGD multiparous mother).*

These revealed that cultural preferences for male children and family mistreatment undermined breastfeeding confidence.

### Determinants enhancing breastfeeding confidence

FGD mothers also described factors that strengthened their breastfeeding confidence. At the time of focus group discussions, participants shared that family support builds confidence to breastfeed their babies.

A 23-year-old primiparous mother said,” *This is my first baby. I gave birth in this hospital, and now I am with my grandmother. I had antenatal care for this baby, and I am confident that breastfeeding exclusively up to six months without complementary food. My grandmother showed me how to breastfeed based on her experience, and my husband also helps me at night. I am happy and confident to breastfeed, and I plan to continue up to two years.”*

This showed that support from family, particularly experienced relatives are essential in building and increasing mothers’ confidence.

Furthermore, prior counseling and health education also played a significant role in breastfeeding self-efficacy. At the time of focus group discussions, participants also shared that prior counseling had built confidence to breastfeed their babies.

*“This is my second baby. I gave birth in this hospital, and now I am with my relative, who is a health extension worker. I had antenatal care for this baby, and I am confident about breastfeeding exclusively up to six months without complementary food. My relative showed me and taught me how to breastfeed based on her experience. I am happy and confident to breastfeed up to two years.” (26-year-old FGD mother).*

These indicated that antenatal counseling and exposure to breastfeeding education positively reinforced self-efficacy.

### Quantitative findings regarding determinant factors

Bivariate logistic regression analysis showed that residence, women’s educational status, recent pregnancy intention, ANC contact, mode of delivery, sex of current baby, having breastfeeding information, breastfeeding experience, and perceived social support were found to be candidate variables (*p* < 0.25) for multivariable analysis.

In multivariable analysis, women’s educational status, ANC contact, breastfeeding experience, and perceived social support were significantly associated with breastfeeding self-efficacy (*p* < 0.05).

In the present study, women with higher educational status (AOR = 1.97; 95% CI: 1.01, 3.83) and (AOR = 3.30; 95% CI: 1.87, 5.85) were more likely to have the ability to confidently breastfeed their baby as compared to those with no formal education. Women who attended 8 or more ANC contacts were 2.24 times (AOR = 2.24; 95% CI: 1.37, 3.63) more likely to be confident in breastfeeding as compared to women who attended fewer than 8 contacts.

Women who had previous breastfeeding experience were three times more likely to have breastfeeding self-efficacy as compared to those who had no previous experience (AOR = 3.59; 95% CI: 2.10, 6.13). Those study participants who had high and moderate perceived social support regarding breastfeeding were 3.23 (AOR = 3.23; 95% CI: 2.02, 6.59) and 2.86 times (AOR = 2.86; 95% CI:1.47,5.55 more likely to have confidence to breastfeed their baby as compared with those women who did not have low perceived social support, respectively (Table 3).

**Table 3.** Bivariate and multivariable analysis of determinant factors of breastfeeding self-efficacy status among postnatal women in Gurage Zone public hospitals, Central Ethiopia, 2025

Breastfeeding self-efficacy status (BFSES) (*n* = 419)					
**Study variables**	**High**	**Low**	**COR [ 95% CI ]**	**AOR [ 95% CI ]**	***P*-value**
**Residence**					
Rural	64	78	1	1	
**Urban**	151	126	1.46 [0.97, 2.91]	1.42 [0.83, 2.42]	0.19
**Women’s educational status**					
No formal education	40	91	1	1	
Primary education	56	38	3.35 [1.92, 5.84]	**1.97 [1.01, 3.83]**	**0.04**
Secondary and above	119	75	3.61 [2.25, 5.78]	**3.30 [1.87, 5.85]**	**0.0001**
**Recent pregnancy intention**					
Unintended	54	65	1	1	
Intended	161	139	1.39 [0.91, 2.13]	0.99 [0.57, 1.73]	0.98
**ANC contact**					
<8 contact	80	113	1	1	
≥ 8 contacts	116	62	2.64 [1.73, 4.02]	**2.24 [1.37, 3.63]**	**0.001**
**Mode of delivery**					
Cesarean section	38	55	1	1	
Instrumental delivery	24	22	1.57 [0.77, 3.21]	1.41 [0.57, 3.47]	0.45
Spontaneous delivery	153	127	1.74 [1.08, 2.80]	1.39 [0.75, 2.58]	0.28
**Sex of the current baby**					
Male	103	74	1.61 [1.09, 2.38]	1.04 [0.62, 1.75]	0.85
Female	112	130	1	1	
**Breastfeeding information**					
No	70	96	1	1	
Yes	145	108	1.84 [1.23,2.73]	1.20 [0.72,1.98]	0.47
**Breastfeeding experience**					
Yes	155	78	4.17 [2.76,6.29]	**3.59 [2.10,6,13]**	**0.0001**
No	60	126	1	1	
**Perceived social support**					
Low	37	82	1	1	
Moderate	61	45	3.0 [1.73,5.19]	**2.86 [1.47,5.55]**	**0.02**
High	117	77	3.36 [2.07,5.45]	**3.23 [2.02,6.59]**	**0.0001**

*Note:* AOR: Adjusted Odds Ratio, ANC: Antenatal Care, BFSE: Breastfeeding Self-Efficacy, COR: Crude Odds Ratio, CI: Confidence Interval, 1: Reference category.

## Discussion

Breastfeeding self-efficacy is a mother’s confidence in her ability to breastfeed her infant. A mother’s belief in her ability to breastfeed strongly determines whether she initiates, persists, and successfully sustains breastfeeding, especially when facing challenges, according to Bandura’s Self-Efficacy Theory (Bandura, 1997). In the present study, the overall prevalence of breastfeeding self-efficacy was 51.3% (95% CI: 47, 56). The finding indicates nearly half of the participants in the study population lacked confidence in their ability to successfully breastfeed, which leads to difficulties in achieving optimal breastfeeding practices. Self-Efficacy Theory indicated that low confidence reduces the motivation and coping ability of mothers, making them more vulnerable to early breastfeeding cessation (Dennis, 2003). The findings highlight the necessity for specific programs, including health education, social support, and counseling, to enhance maternal confidence for better breastfeeding outcomes.

The prevalence observed in this study is in line with a study conducted in the southern Ethiopian region (48%) (Nigussie *et al*., 2025). In contrast, this study is lower than studies conducted in Australia (72%) (Bourne *et al*., 2013), Brazil (82.35%) (Souza and Fernandes, 2014), Thailand (70.3%) (Lynn and Chuemchit, 2024), Indonesia (90.2%) (Hemiyanty *et al*., 2022), Greece (65%) (Brani *et al*., 2024), and Uganda (60.2%) (Nankumbi *et al*., 2019). The possible explanation for this variation may be that healthcare infrastructure differences, combined with limited access to skilled maternal care, strongly affect BFSE. Australia and Brazil maintain advanced breastfeeding support networks with professional lactation experts and organized postnatal programs that many Ethiopian regions lack (Passanha *et al*., 2013; Burns *et al*., 2020; Zegeye *et al*., 2024). In addition to this, cultural beliefs and social norms of countries like Indonesia and Brazil have strong cultural norms supporting exclusive breastfeeding, which enhance breastfeeding self-efficacy as compared to Ethiopian mothers (Agrina *et al*., 2025). Furthermore, the discrepancy may also be explained by variations in the timing of evaluation, sampling techniques, and sample size.

This study found a higher prevalence compared to Brazil (35%) (de Abreu *et al*., 2018) and India (38.8%) (Vaithilingan and Johnson, 2023). The possible explanation may be due to the conduct of the studies, and the characteristics of the participants may explain the differences. The study in Brazil focused only on mothers 12–72 hours after birth and used non-probability sampling, whereas this study focused on the early postpartum period, and the study participants were selected by using the probability sampling method. Moreover, regarding the educational status of the respondents, the higher educational attainment of study participants in the Brazil study was only 16%, as compared to this study, in which 46.3% of study participants had a high educational status (de Abreu *et al*., 2018). On the other hand, the Puducherry, India, study included only first-time mothers, while our study included both first-time and experienced mothers, making it more representative (Vaithilingan and Johnson, 2023). Also, the larger sample size in our study may have given more accurate results than the smaller-sample-size studies.

This study further identified the determinants of breastfeeding self-efficacy among postnatal women in central Ethiopia. The determining factors of breastfeeding self-efficacy included women’s educational status, ANC contact, breastfeeding experience, and perceived social support.

In this study, women’s educational status had a strong association with breastfeeding self-efficacy status; women who completed primary education and secondary or higher were 1.97 and 3.30 times more likely to have the ability to confidently breastfeed their baby, as compared to those with no formal education. *These findings are supported by qualitative findings, as mothers with limited education or first-time mothers often reported low confidence in breastfeeding, particularly when family advice conflicted with health recommendations*. These findings are consistent with studies conducted in Saudi Arabia (Al-Thubaity *et al*., 2023), Greece (Economou *et al*., 2021), Iran (Poorshaban *et al*., 2017), and Indonesia (Titaley *et al*., 2021). The possible explanation might be that as educational status increases, access to information and awareness regarding breastfeeding confidence also increase. Moreover, the evidence shows that, as educational attainment increases, mothers’ ability to handle challenges, make informed decisions, and be more prepared and confident in their ability to breastfeed also increases (Si and Mao, 2024; Nigussiee *et al*., 2025).

In these findings, antenatal care contact was significantly associated with breastfeeding self-efficacy status. Mothers who attended 8 or more ANC contacts were 2.24 times more likely to be confident in breastfeeding compared to women who attended fewer than 8 contacts. *The results are supported by qualitative findings, as mothers who had ANC contact and prior counseling expressed higher confidence in breastfeeding.* These findings are similar to studies conducted in China (Yang *et al*., 2016), Turkey (Topuz *et al*., 2021), and southern Ethiopia (Gizaw *et al*., 2022). A possible justification is that increased frequency of antenatal care contacts enhances women’s access to information, counseling, and guidance on proper breastfeeding practices from healthcare personnel. In addition, breastfeeding education as a routine component of ANC contacts empowers mothers by increasing awareness, dispelling myths about breastfeeding, and boosting breastfeeding confidence (Öztürk *et al*., 2022; Kumari and Jain, 2024; Tello *et al*., 2025). *The qualitative findings indicated that antenatal counseling and exposure to breastfeeding education positively reinforced self-efficacy.*

This finding revealed the strong association between breastfeeding experience and breastfeeding self-efficacy status. Women who had previous breastfeeding experience were three times more likely to have breastfeeding self-efficacy compared to those who had no previous experience. These findings are supported by studies conducted in Malaysia (Johari and Abdul Hamid, 2021), Saudi Arabia (Al-Thubaity *et al*., 2023), China (Yang *et al*., 2016), and Thailand (Lynn and Chuemchit, 2024). The possible reason is that the experiences of breastfeeding positively enhance mothers’ confidence to breastfeed their babies. Furthermore, breastfeeding experiences enhance mothers’ intention to breastfeed and confidence, directly contributing to a strong sense of self-efficacy that supports successful breastfeeding practices (Huang *et al*., 2019). *The qualitative findings indicated that mothers with prior breastfeeding experience reported higher confidence compared to first-time mothers.* This finding is supported by social cognitive theory, which states that prior mastery experiences, such as previous breastfeeding, enhance self-efficacy and mothers’ confidence in breastfeeding (Bartle and Harvey, 2017).

Regarding social support, this study discovered that participants with high and moderate perceived social support for breastfeeding were 3.23 and 2.86 times more likely, respectively, to have confidence in breastfeeding their baby compared with women who had low perceived social support. *The qualitative findings described mothers who had social support from family members as strengthened breastfeeding self-efficacy. These findings suggest that social interventions are important for improving mothers’ confidence in breastfeeding*. This finding is supported by studies conducted in different countries, such as China (He *et al*., 2022), Turkey (Konukbay *et al*., 2024), Brazil (De Sá Guimarães *et al*., 2017), Iran (Mirghafourvand *et al*., 2018), Uganda (Miller *et al*., 2022), and Southern Ethiopia (Gizaw *et al*., 2022). A possible justification for this association might be that mothers who receive social support, especially from healthcare personnel, partners, and families, tend to have higher breastfeeding self-efficacy and greater breastfeeding continuation rate. Perceived social support can be greatly used as a predictor by enhancing self-perception, lowering stress, and boosting sustained confidence in breastfeeding (Can *et al*., 2025; Gopal *et al*., 2025). This aligns with social cognitive theory, which highlights the role of helpful surroundings in shaping health behaviors (Islam *et al*., 2023). The evidence showed that breastfeeding support is a key strategy for improving newborn survival, child health outcomes, and the effectiveness of primary healthcare (Kumari and Jain, 2024; Thacker *et al*., 2025).

These findings show important gaps in knowledge regarding breastfeeding self-efficacy status among mothers in the Gurage Zone, Central Ethiopia. The results suggest that improving health education during antenatal care and postnatal care is essential. Enhancing the quality of antenatal care contact, women’s educational status, the provision of counseling and awareness-creating on breastfeeding, and good social support could significantly improve breastfeeding self-efficacy, which has the potential to reduce neonatal complications and morbidity in the study area. Moreover, the qualitative findings discovered social norms, beliefs, and cultural effects on breastfeeding confidence. These findings have implications for policy and practice in contexts beyond the local setting. Improving breastfeeding self-efficacy globally, especially in culturally distinct areas, supportive social norms shape infant feeding practices. Policymakers are encouraged to integrate culturally sensitive breastfeeding counseling into national maternal and child health guidelines, promote community-based supports, and strengthen health workers to address misconceptions regarding breastfeeding practices. In addition, global health institutions are expected to design interventions on modifiable factors to enhance mothers’ confidence in breastfeeding practices.

## Strengths and limitations of the study

### Strength of the study

In this study, women’s breastfeeding self-efficacy status was assessed using a mixed study design that included both first-time and experienced women. To the knowledge of the investigator, no previous study has been conducted in this setting. Potential biases were minimized by using clear objectives and research questions, pretested questionnaires, training for data collectors and supervisors, a random sampling method, an ideal sample size, and statistical adjustments (multivariable regression) to account for confounding variables. Finally, ethical guidelines were followed to ensure unbiased participation.

### Limitations of the study

In this study, self-reported data on breastfeeding self-efficacy may have introduced recall bias and social desirability biases, leading to possible over- or under-reporting. To minimize this, structured questionnaires with specific prompts and clear pretested tools were used to help participants accurately recall their experiences. Furthermore, facility-based sampling may limit the generalizability of the findings to the wider population. Since this is a cross-sectional study, it does not show cause-and-effect relationships.

## Conclusion and recommendations

### Conclusion

The present findings indicated that 48.7% of women lacked breastfeeding self-efficacy. The educational status, breastfeeding experience, antenatal contact, and perceived social support were significantly associated with breastfeeding self-efficacy. Mothers’ breastfeeding confidence is essential to improve maternal and child health outcomes. Mothers who have high breastfeeding self-efficacy initiate and continue exclusive breastfeeding, which reduces the risk of malnutrition, neonatal infections, and infant morbidities. Enhancing maternal confidence in breastfeeding can lead to progress toward global nutrition and improved child health outcomes. As a result, the study concludes that by intervening in the modifiable factors, postpartum women’s awareness regarding breastfeeding self-efficacy can be increased.

### Recommendations

The following recommendations were given based on the findings to the concerned bodies:

To Gurage Zone public hospitals and health care providers: It is encouraged to provide targeted health education on breastfeeding and its benefits during antenatal care (ANC) contact by using simple, culturally appropriate materials to improve women’s awareness regarding breastfeeding self-efficacy. At the community level, it is encouraged to design community-based awareness campaigns focusing on women with lower educational status and those who have no experience. In addition, encourage health extension workers and community health volunteers in disseminating information at the household and community levels regarding the importance of social support to enhance breastfeeding self-efficacy.

To policymakers and the healthcare system: The study findings show gaps in breastfeeding self-efficacy, ANC contact, and social support. Strengthening the integration of breastfeeding self-efficacy into maternal and child health programs at all levels of the healthcare system aligns with existing maternal and child health policies that promote universal access to maternal health services. Additionally, enhancing maternal confidence in breastfeeding can lead to progress toward global nutrition and improved child health outcomes.

For the researcher: A longitudinal study needs to be done to gain a better understanding of women’s breastfeeding self-efficacy.

## Data Availability

Data that support the findings are available from the corresponding author upon a reasonable request.
